# Capturing and analysing the working conditions of employees with disabilities in German social firms using focus groups

**DOI:** 10.1186/s12889-022-12689-w

**Published:** 2022-03-01

**Authors:** Ilona Efimov, Julia C. Lengen, Ann-Christin Kordsmeyer, Volker Harth, Stefanie Mache

**Affiliations:** Institute for Occupational and Maritime Medicine (ZfAM), University Medical Center Hamburg-Eppendorf (UKE), 20459 Hamburg, Germany

**Keywords:** Job demands, Job resources, Social firms, Employees, Working conditions, Qualitative research, Focus groups, Social enterprises, Occupational health

## Abstract

**Background:**

Social firms – a type of social enterprise – provide job opportunities to people with mental or intellectual, sensory, physical or multiple disabilities who are disadvantaged on the general labour market. Given the limited number of studies on working conditions of employees in inclusive workplaces, the aim of this study was to explore job demands and resources experienced by employees with disabilities in German social firms.

**Methods:**

Three focus groups were conducted between September and October 2020 with 14 employees with disabilities from social firms in the catering and cleaning sector in Germany. The Job Demands-Resources model was used as a theoretical basis for developing the semi-structured interview guide. Audiotaped data were transcribed verbatim, analysed deductively and inductively using the qualitative content analysis according to Mayring.

**Results:**

The results show that employees of social firms experience specific job demands and resources regarding work content, work organisation, social relations and work environment. Job demands were mainly reported with respect to work organisation, e.g. high workload, time pressure or challenges in collaboration, whereas social relationships with colleagues and supervisors were most frequently mentioned as important resources at the workplace.

**Conclusion:**

First exploratory study results on the working conditions of employees in social firms in Germany were obtained. Given the pivotal importance of employment for people with disabilities, the identified job demands and resources of this study highlight the relevance of a healthy workplace, especially for employees in social firms. Future interventional research is needed regarding the development, implementation and evaluation of workplace health promotion measures in social firms.

**Supplementary Information:**

The online version contains supplementary material available at 10.1186/s12889-022-12689-w.

## Background

In 2019, about 7.9 million people representing 9.5% of the population were living with a severe disability in Germany, whereof about 40% of them were at working age between 25 and 64 years [1]. For people with severe disabilities there are several barriers on their way to gainful employment [2, 3]. Consequently, the employment rate of people with severe disabilities is considerably lower compared to the general population [4]. In Germany, only 46.9% of the people with disabilities in contrast to 75.2% of the general population do have a job [5]. Considering positive effects of work for recovery of employees with mental illnesses [6–10] and social integration [11, 12], high unemployment rates seem to be particularly challenging. Enabling people to gain and maintain employment – for example in social firms – can have a more positive impact than other medical or social interventions [13] and can contribute to a successful inclusion [14]. Employment of people with disabilities in social firms is therefore recommended, especially from a social and health perspective.

According to the United Nations Convention on the Rights of Persons with Disabilities (CRPD), social firms, as inclusive enterprises, foster the equal inclusion of severely disabled people in the general labour market by creating safe and healthy working conditions as well as the opportunity for these people to earn a living through freely chosen work (Article 27) [15]. Social firms were initially developed in Italy in the 1970s and led to the foundation of many other companies in other countries [16]. In Germany they provide severely disabled people with mental or intellectual, sensory, physical or multiple disabilities who are disadvantaged on the general labour market job opportunities (§ 215, section 2, Book Nine of the German Social Code (SGB IX)), work-accompanying support and an occupational setting respecting their individual needs [6]. Thereby, social objectives are primarily followed and profits are reinvested back into the company or community [9]. If required, initial or vocational training or opportunities to participate in extra-company measures are also offered. Social firms also provide support for placement in other companies on the general labour market (§ 216, section 2, Book Nine of the German Social Code (SGB IX)). In addition, social firms receive financial support for expanding, modernizing or equipping their facilities according to the needs of severely disabled employees as well as for providing accompanying assistance in working life (§216 and § 217, Book Nine of the German Social Code (SGB IX)). In Germany, social firms have to employ 30 to 50% of people with disabilities (§215, section 1 and 3, Book Nine of the German Social Code (SGB IX)). There are approximately 900 social firms employing about 13,550 employees with disabilities in Germany [17], basically located in the sectors catering (18%), gardening and landscaping (11.4%), industrial production (13.4%), facility management (11.2%), trading (12.4%) and handicraft (12.6%) [18].

Relevant factors influencing employees’ health in social firms have been investigated in some countries (especially in Canada, the United Kingdom, Australia and Italy) [19]. Kordsmeyer et al. (2020) gave a comprehensive overview of the working conditions and their impact on employees’ wellbeing and other health and work-related outcomes [19]. According to current state of research, the design of job tasks, expectations of supervisors, work environment, conflicts with co-workers, disregarded comfort in social interactions, heavy workloads, time pressure or organisational constrains were found as possible job demands of employees with disabilities working in social firms [13, 14, 20–27]. Furthermore, social and organisational support, social events, feedback, tolerance for errors, schedule flexibility, flexible workloads, structured work tasks, training, job security and participation at the workplace were identified as possible job resources of employees in social firms [6–8, 11, 13, 14, 20–36]. Thereby, most existing studies focused mainly on job resources like flexible working arrangements or social support and relatively few on job demands in social firms. Given the pivotal importance of employment for people with disabilities [37] and the lack of comparable studies to date in, e.g. German-speaking countries [19], there is a need for further exploratory research.

### Theoretical framework

The Job Demands-Resources model (JD-R model) by Bakker and Demerouti (2007) served as an occupational health psychological theoretical framework for this study [38]. The well-evaluated model classifies work characteristics into demands and resources. Job demands relate to aspects that involve unfavourable psychological and/or physical efforts and therefore entails psychological and/or physiological costs. An accumulation of these job demands can lead to exhaustion and health problems. In contrast, job resources have motivational potential and relate to beneficial aspects of the job (for instance by leading to high work engagement or low cynicism). Perceived job resources may reduce potential job demands at work and be conducive to personal development, motivation and work engagement [38, 39]. The JD-R model is suitable as its flexibility facilitates the investigation of diverse aspects of an occupational setting [40, 41].

The categorisation of relevant work characteristics according to the Joint German Occupational Safety and Health Strategy (GDA) was applied for the analysis of respective job demands and resources: work content, work organisation, social relations and work environment [42]. These four categories are based on a broad systematic research on the relationship between working conditions and mental health carried out by the German Federal Institute for Occupational Safety and Health (BAuA) [43].

### Study aims and research question

Based on the underlying assumption of Bakker and Demerouti (2007) that any occupational activity exhibits its own job demands and resources [38], the aim of this study was to gain exploratory insights into job demands and resources of employees with disabilities in German social firms according to the criteria of the Joint German Occupational Safety and Health Strategy (GDA) [42]. Given the limited number of studies on working conditions of employees in social firms [19], there is a need for exploratory research.

Therefore, the study aimed to provide answers to the following research question: *What job demands and resources do employees with disabilities in social firms experience with regard to their work tasks, work organisation, social relations or work environment?*

## Materials and methods

### Study design

The present study was part of a three-year research project aimed at developing evidence-based health promotion interventions for social firms. As part of a triangulation analysis, three focus groups were conducted in German social firms between September and October 2020 with 14 employees with disabilities. This qualitative research method was used to gain an in-depth understanding of the target groups’ job demands and resources [44]. Focus groups were considered to be the most appropriate method to explore the subjective perceptions, experiences and opinions of employees with disabilities. In addition, this method is particularly suitable for examining people’s different perspectives [45]. The consolidated criteria for reporting qualitative research (COREQ) were taken into account for reporting the study design and results (see Additional file 1: COREQ-Checklist) [46].

### Participant selection

The study took place in collaboration with social firms in northern Germany. As gatekeepers, the social firms offered the research team the opportunity to recruit and interview employees with disabilities in their companies. Prior to the focus groups, three researchers carried out participatory observations in social firms [45]. Thereby, researchers and participants got to know each other, the research team was able to establish trust and explain their scientific and personal background for conducting the study. Subsequently, the researchers invited the employees to participate in focus groups. For this purpose, a flyer in easy language – handed out by the supervisors – was offered as well as the possibility to ask the moderating researcher (JL) questions about the research project.

Participants were eligible for a focus group, if they met the following criteria: 1) a severe disability; 2) a paid job in a social firm; 3) older than 18 years and 4) proficiency of the German language (including sign language). To create heterogeneous focus groups, participants were selected through convenience sampling, taking gender, age, type of disability and department into account. In order to give participants a feeling of protection and uninhibited speech, the focus groups were not mixed across company boundaries. Pre-existing groups were chosen to allow employees to report experiences in a familiar setting and to develop improvement suggestions for the target group itself [45]. Due to the gatekeeper function of supervisors, the researchers had no insight into the total number of employees who were interested in participating in the study. Based on feedback received by supervisors, the reasons for not participating in the study were a lack of motivation/willingness to participate, fears of not being understood or saying something wrong, a lack of confidence, language barriers (German language) or sickness absences.

### Data collection

One focus group was held at the workplace of the participating social firms and two focus groups were held in conference rooms outside the companies. Each group consisted of three to six employees and two researchers. The focus groups were conducted in German and lasted approximately 90 min. No focus group was repeated. The focus groups were moderated by two female researchers of the research team: One researcher (JL), a health scientist with experience in qualitative research and practical experience with the target group, moderated the focus groups based on the interview guide. Another health scientist (ACK) assisted and co-moderated, visualized shorthand notes of discussion results and verified interpretations with participants [44]. A semi-structured interview guide with open questions was developed to guide the moderator, to focus on the research questions, to ensure comparability of the focus groups and to increase reliability (Table 1) [45]. The comprehensibility of the interview guide regarding plain language was aligned with a cooperating provider of health promotion offers for people with disabilities. A pre-test was performed beforehand with a person working in a comparable setting. The focus groups started with the researchers explaining the study aim, introducing into the conversational rules and code of conduct. Postcards with occupational health and safety related comics were handed out to stimulate conversation about participants’ job demands and resources [45]. The comics were provided in agreement with a related WHP project to be used methodologically in the focus groups [47]. After the group interaction, a short questionnaire was handed out to all participants to obtain demographic data.

**Table 1:** Parts of the interview guide

**Job resources**	
• What do you like most about your job?	
• Who or what supports you at work?	
• What helps you to get the job done?	
**Job demands**	
• What is particularly difficult/exhausting/annoying at work?	
• What makes you feel uncomfortable at work?	
• What makes work difficult for you?	
• Is there anything that annoys/frustrates you at work?	
• What is particularly stressful at work?	

### Data analysis

With regard to data analysis, all focus groups were audiotaped and the data was anonymized and transcribed verbatim according to Kuckartz (2016) resulting in over 61 pages of data [45, 48]. The transcripts were analysed deductively and inductively using the qualitative content analysis according to Mayring [49]. This well-validated method was chosen, as it follows a systematic and rule-based approach using a category system focusing on the semantic content of the data [49]. For both analysis steps the software MAXQDA Plus for Qualitative Data Analysis (Version 20.0.6, 2020, VERBI GmbH, Berlin, Germany) was used. The data transcription and analysis was carried out by two researchers (JL and IE) without involving the participants. The category system’s main categories were derived in a deductive way referring to the interview guide and theoretical framework. As underlying theory, the JD-R model by Bakker and Demerouti (2007) and the job criteria (work content, work organisation, social relations and work environment) of the Joint German Occupational Safety and Health Strategy (GDA) were consulted [38, 42]. Further sub-categories were established in an inductive way [49]. All data were coded by a health scientist (JL) and occupational psychologist (IE) and double-checked by two researchers (ACK, IE). Disagreements in the coding and grouping process were discussed in the research team until consensus was reached. Representative quotes of the focus groups were translated and presented to illustrate the findings.

### Ethical considerations

All participants signed an informed consent form, which was additionally handed out in plain language with visual material at the start of each focus group. Written and oral information was provided to all participants on data protection, the confidentiality and anonymity of study results. All participants were literate and received assistance from research assistants when needed to understand and sign the informed consent form. The study was conducted in accordance with the Declaration of Helsinki and approved beforehand by the Ethics Committee of the University Medical Center Hamburg-Eppendorf, Germany (LPEK-0051).

## Results

### Participants

Three focus groups were conducted with 14 participants in total. Each focus group involved employees with disabilities working within the same social firm. Two focus groups consisted of employees who were employed in the catering sector (food and beverage service activities [50]) and one in the cleaning sector (cleaning activities [50]). The participants were equally male and female. Most employees were either 18–29 or 40–49 years old. The majority of participants have been employed for more than three years (Table 2). Employees performed work activities in the kitchen, scullery, customer service, cleaning of facilities, housekeeping activities, administrative tasks or supporting activities.

**Table 2:** Characteristics of study participants (*n* = 14)

Characteristics	***n***	%
**Gender**		
Male	7	50.0
Female	7	50.0
**Age group (years)**		
18–29	5	35.1
30–39	1	7.1
40–49	6	42.9
50–59	1	7.1
60 +	1	7.1
**Period of employment**		
< 1 year	2	14.3
1–3 years	1	7.1
> 3 years	11	78.6
**Sector**		
Catering	11	78.6
Cleaning	3	21.4

### Job demands of employees working in social firms

In total, nine sub-categories were identified inductively. These were assigned to the four main categories formed deductively according to work characteristics defined by the Joint German Occupational Safety and Health Strategy (GDA) [42]. In the following, these categories are described in detail: stressful work tasks, high workload and time pressure, unfair distribution of work and challenges in collaboration, working hours, insufficient reward, interfering or gossiping colleagues, pressure from supervisors, physical effort and noise.

#### Work content

##### Stressful work tasks

Several employees assessed it as burdensome to work alone and bear the associated responsibility autonomously. It was described as especially problematic in case of absenteeism of personnel and in combination with overcharging workload, as it leads to stress and high requirements on employees’ coping strategies.

*“You’re an absolute lone wolf. There is no staff there.” (Employee #6, male, catering sector)*

Single interviewees also mentioned repetitive work tasks as negative.

*“It’s all just a circle, so to speak. The constant cleaning […]. The up and down, again, and everything again, and again, again. These are negative things.” (Employee #2, female, cleaning sector)*

Cleaning activities were partly associated with feelings of disgust as illustrated in an interviewer’s example of sorting out expired food.

*“Yes, we also threw away everything from over there, it just had to go. Even in the refrigerator, such old things, me and [name of colleague] did it together. But that was just disgusting to see.” (Employee #3, female, cleaning sector)*

#### Work organisation

##### High workload and time pressure

The most present job demand in the focus groups was a high workload partly due to staff shortage. This was associated with overload.

*“But I’m overwhelmed at work. I have so much/ I can’t do it alone.” (Employee #8, male, catering sector)*

Several employees reported a lot of stress at work and described working under time pressure as demanding.

*“I mean, there’s so much stress in the kitchen. We work against the clock like that, don’t we?” (Employee #11, male, catering sector)*

*“So to speak, to work on time and to make sure that you work as little overtime as possible. But I’d also like to get everything done, and especially if you’re a little slower, right?” (Employee #2, female, cleaning sector)*

Few interviewees expressed their wish for a smaller amount of work. One interviewee described a more extended need for recovery resulting from stressful working days. As a side effect, time pressure was described to impede healthy behaviours at the workplace, such as complying the health and safety instructions for lifting heavy weights.

*“Yes, we also have such a risk analysis, but also/ yes, you just have to bear it in mind when you’re under stress, right?” (Employee #6, male, catering sector)*

##### Unfair distribution of work and challenges in collaboration

Some interviewees described an unequal distribution of tasks, which was partly perceived as unfair. It was reported that some colleagues, e.g. take many (smoking) breaks, work very slowly, are unmotivated or do not fulfil their work assignments. This was perceived as burdensome, especially in intensive working phases.

*“There are also other problems. He’s slow, he does everything slowly. Not just smoking!” (Employee #9, female, catering sector)*

*“Yes, I know the people. I don’t name them, but they can work well. But they don’t work. They’d rather hang out somewhere and go to the bathroom or something, they’re wasting time. Or go smoke.” (Employee #14, male, catering sector)*

A lack of adaptability among some new colleagues, sometimes even in terms of misconduct, was also described as problematic. One recently employed interviewee outlined a turbulent initial phase in the company.

*“It’s not as bad as it was before. At first, I actually could understand it, where I came here, I also used to think, ‘What’s going on here?’ First of all, calm down.” (Employee #1, female, cleaning sector)*

Another factor reported as a challenge to collaboration was the inadequate quality of work and motivation of certain employees.

*“There are two employed, [...] they also sometimes say, ‘I don’t feel like it.’ [...] I witness that. They just say, ‘Let [name of colleague] do it alone’, and I find that a bit irritating, too.” (Employee #14, male, catering sector)*

Especially in combination with unclear responsibilities or dependencies between colleagues in terms of intermediate working steps it was described as problematic for work processes and demanding for employees.

*“I’m always dependent on others help [...] and that sometimes […] doesn’t work so well.” (Employee #8, male, catering sector)*

##### Working hours

Early working times seem to affect employees differently. Some remarked that getting up and starting work early contributes to greater exhaustion.

*“I started at 4:45 in the morning. I get up at three o’clock. Then I am slightly exhausted.” (Employee #9, female, catering sector)*

*“Oversleeping gets me upset [laughing]. Yes, when you can’t sleep through the night, it always happens. Or when you don’t hear the alarm clock.” (Employee #13, female, catering sector)*

Another employee described that they got used to early working hours.

*“We always start at 6 o’clock, 7 o’clock. [...] We got used to it already.” (Employee #11, male, catering sector)*

##### Insufficient reward

A few employees perceived the often payed minimum wage as too low for their job performance. They wished for more reward, e.g. in form of a monetary bonus for long-standing workers or subsidised sports courses.

*“I wish they paid a little better. Well, I only get the minimum wage here and that’s exhausting work and that could be praised. Especially when you’ve been here for 10 years.” (Employee #8, male, catering sector)*

*“Or, for example, that something else is being paid for. Sport or something. That also serves one’s health. [...] If it’s not a lot of money, then at least some other kind of loan.” (Employee #6, male, catering sector)*

#### Social relations

##### Interfering or gossiping colleagues

Complaints, orders given by colleagues, unrequested interference into team issues (such as work and break times) or gossiping from colleagues were rated as demanding. In response, employees explained to avoid private conversations when concerned colleagues are present.

*“That’s not our team, but [...] some of them always try to boss us around the way our work is to be done sometimes, and we don’t need that.” (Employee #3, female, cleaning sector)*

*„Sometimes there are situations where [...] sometimes people interfere a bit and [...] think they are better, know something better, although they do not even belong to this area.” (Employee #1, female, cleaning sector)*

Therefore, it was emphasized, that above all, the discretion of the supervisor is highly important in order to establish a trustful relationship.

*“There [...] you also have to know that anything you say to your supervisor doesn’t somehow get to other people, so that’s/ sometimes there’s a lot of gossip and then you don’t know who was talking or something like that. And then/ I personally just withdraw from it.” (Employee #6, male, catering sector)*

##### Pressure from supervisor

The interviewees described only very few situations in which the supervisor had exerted pressure on them. However, one situation was perceived negatively in which a supervisor criticized the employee’s work process, which was also experienced as emotionally destabilizing by the employee.

*“So I had a/ a very intense conversation that was a bit (...) not so nice. [...] That was already/ for me, that was already heavy [...]. The situation, that really tore the ground from under my feet for that day.” (Employee #6, male, catering sector)*

*“When the boss puts so much pressure on me, I’m loud too. I also say what’s inside me.” (Employee #14, male, catering sector)*

It was also noted that pressure was not only communicated verbally, but could be transferred to employees when supervisors were stressed.

*“Especially when the boss is stressed, I think it spills over.” (Employee #6, male, catering sector)*

#### Work environment

##### Physical effort

Various workplaces involved physical strain such as walking around and carrying heavy weights.

*“I just walk all day long around, around, around.” (Employee #14, male, catering sector)*

*“I don’t know how many litters fit in there. But I think it was already forty, fifty kilos that we lifted. That was quite a lot.” (Employee #6, male, catering sector)*

##### Noise

The kitchen was generally described as a noisy working environment, which was perceived as stressful in some cases. One employee explained that he felt disturbed by the noise of the machines, the ventilation system as well as a very communicative, loud speaking colleague. The noise level was described as a stress factor that also affected their health.

*“Yes, people often talk there [...] and then the machines are loud, the ventilation is loud. It’s annoying. You notice that in the evening, when you come to rest, that it echoes.” (Employee #8, male, catering sector)*

*“I’m a person who needs to rest, yes. It annoys me. But maybe that’s just me, I don’t know. Others deal with noise differently. [...] Yes, during the week it is somehow a stress factor that affects one’s health.” (Employee #8, male, catering sector)*

Still other employees reported getting used to the noise of their working environment.

*“I mean, the kitchen is loud. [...] I mean, even when the music isn’t playing and when we’re under stress/ our voices are also loud so that we can understand each other better and no misunderstandings arise. [...] But we’re so used to it, we/ it all seems so normal.” (Employee #11, male, catering sector)*

### Job resources of employees working in social firms

In total, 11 sub-categories were identified inductively. These were assigned to the four main categories formed deductively according to work characteristics defined by the Joint German Occupational Safety and Health Strategy (GDA) [42]. In the following, these categories are described in detail: fulfilling work tasks, moderate workload, established work processes, communication, flexibility and autonomy in organizing break times, reliable payment, team cohesion, relationship with the supervisor, working atmosphere, occupational safety and snacks and drinks for team meetings.

#### Work content

##### Fulfilling work tasks

The work tasks in the service were described as meaningful, because clients appreciated clean premises or tasty served dishes. Accordingly, customer contact was enjoyed by employees due to appreciative and positive feedback.

*“That’s the ultimate gratification. So when they say that it was delicious.” (Employee #6, male, catering sector)*

*“That’s just what I enjoy. Being there for people.” (Employee #5, female, catering sector)*

The employees reported to be proud after finishing the assigned tasks.

*“I’m glad I can do it/ have done it.” (Employee #2, female, cleaning sector)*

Moreover, the interviewed employees described an extended task spectrum, as for example some office tasks added to their regular cleaning tasks, as beneficial and diverse.

*“Yes [it bothers me that it’s often the same]. [...] That I also have to do the office work in addition to the cleaning, because others can’t, won’t do it.” (Employee #2, female, cleaning sector)*

#### Work organisation

##### Moderate workload

Several employees stated that they were able to cope with stressful work periods when relaxation phases followed.

*“I am now working full-time again. [...] And then the change to working full-time again with no relief [...]. That was quite an adjustment.” (Employee #6, male, catering sector)*

One employee described that an adjusted workload appropriate for the employee’s individual capacities resulted in less pressure and higher well-being compared to a higher workload in the past.

*“Less work. Now we are more people, before we were really few people, less workers and then I had to work a lot.” (Employee #9, female, catering sector)*

The agreement of an appropriate workload for specific needs, based on individually adapted work packages, was also experienced positively. While one employee deemed checklists as helpful for reminding, another one experienced long lists of all working tasks as burdensome and creating pressure and preferred a small-scaled allocation of working tasks.

*“That’s when she was stressed because she knew what to do. She knew everything. But here she doesn’t know, here she always has to ask [supervisor] or [other supervisor]: ‘What should I do, what should I do?’.” (Employee #14, male, catering sector)*

##### Established work processes

Collaboration in a well-coordinated, experienced team, having a clear allocation of tasks and responsibilities, well-functioning work processes and communication patterns were experienced as beneficial. These circumstances were described to be often related to long-standing collaborations.

*“We also work together for a long time. We know what makes the young man go. And this helps too, right?” (Employee #14, male, catering sector)*

Employees also valued the experience of being involved in designing the work environment, for example when improving work processes.

*“I made sure that each department has several ones [safety displays] [...] so that we could have our peace and quiet. That there’s no longer such a fuss, because we don’t always have to wait.” (Employee #1, female, cleaning sector)*

##### Communication

Good communication, stimulated by regular, scheduled team meetings, was reported to be beneficial, e.g. by solving problems, whereas supervisor attendance was just partly desired. In case of conflict, several employees favoured talking to each other directly and honestly to find solutions over involving the supervisor or other people.

*“Actually, we usually [...] sit down together for ten minutes on Friday and then any problems [...] that really don’t work anymore, we talk about it and then it’s already solved on Monday. [...] Then we just talk to the boss, then it gets cleared up and then everything is good again.” (Employee #11, male, catering sector)*

Participants expressed that they wish for productive collaboration in their daily work routines, so that not only the supervisor’s announcements are complied, but also collegial agreements are respected. Collegial agreements, also in form of short briefings for allocating and handing over work, were considered as valuable. Informal conversations about personal issues between colleagues were described as important for collaboration.

*“That it doesn’t spill over somewhere else. First talk, then as a team with one another, even if it doesn’t affect you. Nevertheless, one simply tries to listen. That you just talk about it honestly from time to time.” (Employee #1, female, cleaning sector)*

##### Flexibility and autonomy in organizing break times

Some interviewees appreciated the possibility to organize break times in a flexible and individual manner.

*“Today I’m not taking a break, I’m just cleaning through here. No break today, so I can just leave a little earlier.” (Employee #3, female, cleaning sector)*

##### Reliable payment

Although the amount of the salary was partly experienced as unsatisfactory, an employee appreciated the reliable regular payment in his job.

*“That you get paid on time. That’s always good. You know you’ll get your money.” (Employee #8, male, catering sector)*

#### Social relations

##### Team cohesion

In all three focus groups a supportive team was mentioned as an important resource. Social support from colleagues was experienced as helpful in overwhelming situations or in situations when employees felt helpless or stressed. Understanding and social support provided by colleagues, e.g. in case of spontaneous absence from work or need for a break, helped employees to regenerate from work.

*“To have the courage when you’re not in a good mood or to somehow say, ‘Hey, I’m not feeling well today.’ (...) And then there’s already support from the others.” (Employee #6, male, catering sector)*

One team described themselves as a family. They had spent a lot of time together, laughed a lot, collaborated and got along with each other well. Kind colleagues were described to make it easier to get up early and thus promote going to work.

*“So I really have to admit, our team is truly just like a family.” (Employee #11, male, catering sector)*

Another team described a strong cohesion within the team, which protects and stands up for each other in difficult situations. Integrity and trust were mentioned as resources in the working context.

*“Of course, I also protect my colleagues, because we work together as a team.” (Employee #3, female, cleaning sector)*

Furthermore, it was reported in several focus groups that colleagues arranged to meet outside work time. This was associated with a good mood and joyful anticipation.

##### Working atmosphere

A positive, friendly working atmosphere and harmonious collaboration without hostility or disputes were experienced as beneficial for health.

*“Here it’s positive, here’s everyone nice.” (Employee #3, female, cleaning sector)*

Participants of one focus group described their harmonious collaboration in terms of having fun at work together, laughing, chattering and teasing each other in a friendly way – partly also with supervisors. A pleasant working atmosphere was found to be a cause of good mood and made working hours pass quickly. Also listening to music and combining cleaning tasks with dancing was highlighted.

*“So just before work ends, actually starting at three o’clock, when we are cleaning the kitchen, we turn on the music full blast and then/ Then we really dance and work, like that. So that’s just how it is at our place.” (Employee #11, male, catering sector)*

Several interviewees described their joy in working together and attested a higher well-being when working in a team. Teamwork was also experienced to contribute to work being perceived more successful, and it motivated and made work easier, even when the workload was high. Some participants described that colleagues also used to remind them of safety instructions in case they forgot them during high workloads and stressful experiences.

*“There’s usually a free trolley there, so we take it with us. But under stress you can forget that sometimes/ but we already pay attention to it. We say, ‘Why don’t you take a trolley?’.” (Employee #6, male, catering sector)*

*“Working together. I think that’s really great. Because you can’t manage everything on your own. You always have to work in a team, and then you can accomplish more.” (Employee #14, male, catering sector)*

Employees with disabilities valued the strengthening and accepting way they were treated in the social firm. Respect towards the diverse group of employees, listening, an appreciative and a non-discriminatory interaction were mentioned as positive aspects. Long-standing collaboration with colleagues was reported to support the mutual acceptance of individual difficulties at work. Furthermore, a positive and patient way of dealing with mistakes combined with a calm communication by supervisors were appreciated.

*“That one also empowers the people with disabilities and that one is there for them and accepts them, because they also do good work.” (Employee #7, male, catering sector)*

##### Relationship with the supervisor

A good relationship with the supervisor was considered as important. Employees valued it positively when their supervisor listens to them, treats them respectfully, gives them feedback and the opportunity to express wishes. Supervisor support in general was mentioned by several employees as a resource. Employees described being able to contact their supervisor on bad days, for health-related problems or impairments as well as a support or mediator for work conflicts.

*“There is someone I can talk to about it.” (Employee #5, female, catering sector)*

Many employees described an appreciative interaction, verbal rewards and compliments for good work as well as passing on feedback from clients by their supervisor as beneficial.

*“Great place here to be praised.” (Employee #3, female, cleaning sector)*

In general, social firms were described as a setting in which comparatively less pressure is exerted on employees by supervisors.

*“Years ago I also worked in different companies [...]. There was always pressure and I became a bit confused and then (...) I had a problem and then I got a disabled persons pass. Then I started here. [...] In our company it’s not like that. [...] Quite seldom. Yes, that was just/ because I experienced that before. But [...] here, I haven’t experienced anything like that a lot.” (Employee #14, male, catering sector)*

#### Work environment

In case of transporting heavy weights at work, appropriate equipment (e.g. lifting aids or trolleys) and guidelines, like occupational safety instructions (e.g. lifting heavy weights only in pairs) were named as other resources.

*“Always lifting with two men, then nothing will happen. No lumbago.” (Employee #4, male, catering sector)*

As a sign of appreciation, employees highly valued provided snacks and drinks for team meetings.

Table 3 summarizes identified job demands and resources of employees working in social firms.

**Table 3:** Overview of job demands and resources of social firm employees

Job criteria	Job demands	Job resources
**Work content**	**Stressful work tasks:** • Self-responsibility, working alone • Repetitive work tasks • Feelings of disgust	**Fulfilling work tasks:** • Meaningful tasks • Customer contact • Being proud of accomplished work • Task variance
**Work organisation**	**High workload and time pressure:** • Overload due to staff shortage • Need for recovery from stressful working days	**Moderate workload:** • Adjusted workload to individual capacities • Adequately assigned work packages • Alternating tension and relaxation phases
	**Unfair distribution of work and challenges in collaboration:** • Unequal allocation of workload • Unestablished working processes, unclear responsibilities • Perceived lack of motivation, inadequate quality of work of certain colleagues	**Established work processes:** • Clear allocation of tasks and responsibilities • Participation
		**Communication:** • Regular, scheduled team meetings • Solving problems within the team • Collegial agreements • Informal conversations
	**Working hours:** • Early working times	**Flexibility and autonomy in organizing break times**
	**Insufficient reward:** • Minimum wage too low for job performance	**Reliable payment**
**Social relations**	**Interfering or gossiping colleagues:** • Complaints • Orders given by colleagues • Unrequested interference into team issues	**Team cohesion:** • Social support from colleagues • Integrity and trust • Private meetings in leisure time
	**Pressure from supervisors:** • Verbal communication • Stress of supervisor can be spilled over to employees	**Relationship with the supervisor:** • Supervisor support • Mediator for work or team conflicts • Appreciative, respectful interaction, verbal rewards, feedback
		**Working atmosphere:** • Harmonious collaboration • Having fun at work (together) • Motivating teamwork • Respect • Appreciative, non-discriminatory interaction • Mutual acceptance of weaknesses • Patience in dealing with mistakes
**Work environment**	**Physical effort:** • Walking around a lot • Carrying heavy weights	**Occupational safety:** • Appropriate equipment and guidelines
	**Noise** • High noise level in the kitchen	**Snacks and drinks for team meetings**

## Discussion

To our knowledge, this study is the first to provide empirical results on working conditions of employees with disabilities in German social firms. In our focus groups, employees were asked about their job demands and resources. Referring back to the JD-R model [38], GDA criteria [42] and our study aims, the results show that employees from social firms experienced specific job demands and resources concerning work tasks, work organisation, social relations and work environment.

### Job demands

In terms of *work content*, some employees described repetitive tasks and taking responsibility at work as stressful. More specifically in the cleaning sector, some job-specific tasks were associated with feelings of disgust. Lanctôt et al. (2012) also found that the perception of work tasks is highly individual among employees with disabilities (e.g. preference for repetitive or more challenging job tasks) and thus attention should be paid to balancing favourable and unfavourable tasks [51].

Job demands regarding *work organization* were discussed in the focus groups in particular. Several employees reported a high workload and time pressure due to staff shortage, early working hours and perceived an unfair distribution of work and challenges of collaboration in their team. Previous studies have also shown that a high workload, time pressure and unfavourable working hours were experienced as especially demanding by employees in social firms [21, 25–27]. They identified negative influences of high levels of organizational constraints on employees’ work productivity, job satisfaction, and motivation to hold the job [25–27]. Similar to our findings, dissatisfaction with working hours were also reported in other studies, in terms of reduced working hours for those holding disability benefits, irregular changes of working hours or late afternoon shifts [14, 21, 30]. Moreover, the distribution of working hours among employees was described to may lead to interpersonal conflicts [22]. Furthermore, some employees of the focus groups considered their minimum wage to be too low for the work performed and wished for an additional form of remuneration. To date, research has only been able to show a strongly supportive effect of payment and job security of employees in social firms [8, 20, 21, 28, 32, 33]. However, individual studies indicated that the majority of employees earned above minimum wage but had little prospects of earning higher wages [14] as well as had limited advancement possibilities [21, 32, 33].

*Social relations* at work were also perceived as demanding when colleagues complained a lot, gossiped, interfered in team matters unsolicited or gave non-authorized orders, as well as when supervisors put too much pressure on their employees. This pressure was experienced to be exerted directly via verbal communication or indirectly via the supervisor’s stress spilled over to employees. Similar to present results, a previous study found that less conflict among colleagues was important for building social relationships and conducive to perceived quality of work life [20]. Other studies found that social relationships at work were perceived as straining by employees in social firms when they felt they were becoming a burden to the team due to not completing tasks in time [21] or were perceived as patients rather than employees by customers with persistent stereotypes [23]. Several studies showed that insufficient attention to specific social needs of employees regarding their comfort with social interactions may arise as a job demand [14, 22, 23]. Moreover, our findings regarding pressure of supervisors coincide with the results of previous studies. Thus, supervisors should adjust their expectations regarding the work pace and put less pressure or demands on their employees [14, 20, 22]. In contrast, a qualitative study of employees with severe mental illnesses found psychological harassment by supervisors to disfavouring perceived quality of work life [20].

Lastly, specific aspects of the *work environment* were stated by the participants as distressing. The amount of walking and carrying heavy weights was experienced as physically tiring. Especially in the catering sector, the acoustic exposure to noise was partly reported to be challenging. Similar job demands due to noise or physical constraints were also demonstrated in a previous qualitative study from Canada [20]. Another study reported physical constraints due to cleaning in hot offices without air conditioning by employees working in the cleaning sector [21]. Our study could not replicate this finding, e.g. due to differences in climatic conditions in northern Germany.

So far, only little research investigated job demands of employees working in social firms [19]. Our study complements the current state of research, especially with in-depth results on perceived stressful aspects of work organisation and social relations.

### Job resources

With respect to *work content*, several employees reported in the focus groups that they enjoyed meaningful tasks, customer contact, task variance, and felt proud of accomplished work. Existing research confirms that a perceived meaningfulness of work tasks is fundamental for developing workplace identity and is reflected, e.g. in customer demand or payment [6, 8, 11, 29, 30]. As shown in our results, a perceived (additional) responsibility at work was also described in other studies as conducive to job satisfaction [21, 28, 32]. In addition, other studies were able to demonstrate that skills development was considered relevant for future career steps, e.g. for the transition to other companies in the general labour market [14, 20, 22]. Overall, training opportunities for employees in social firms were found to be beneficial for developing a sense of expertise [13, 20, 24, 29, 32] and were associated with increased job tenure [24].

In the context of *work organisation*, several employees mentioned a moderate and adjusted workload according to individual capacities, established work processes and team communication as well as flexible and autonomous break scheduling as job resources. Thereby, alternating tension and relaxation phases, a possibility of participation at the workplace and reliable payment were experienced positively. In terms of good team communication regular, scheduled team meetings but also informal conversations had enabled problems to be solved within the team and agreements to be reached in a collegial manner. Similarly to our results, previous studies found that flexibility in performing work tasks [6, 22, 30, 32] as well as adjusted tasks to individual skills, capacities and interests is beneficial for employees [8, 14, 31]. Furthermore, flexible work arrangements in the context of scheduling were found to have a positive impact on performance, well-being, job satisfaction [21, 32], and job tenure [24]. In general, consistent and structured work activities have been shown to promote, amongst others, self-efficacy, community participation and work identity [6–8, 11, 21, 23, 32] or to reduce stigma of mental illness [36]. Equally as in our study findings, previous research has highlighted the relevance of participation at work for employees from social firms, e.g. in decision-making processes [11, 28–31]. Although supportive social relationships at work have been most frequently mentioned in current research [19], there are no comparable results on communication processes in teams of social firms, e.g. related to problem solving. However, other findings on the positive perception of regular salary payments have also been shown in previous studies as they were associated with financial independence as well as social participation [21, 22, 28, 30, 32]. Current research demonstrates that payment and job security have generally been found to be supportive for, inter alia, employees’ job satisfaction and well-being [8, 20, 21, 28, 32, 33]. Additional benefits, e.g. paid annual leave, public holidays or pension also increased perceived job security and thus job satisfaction [21, 32].

*Social relations* with colleagues or supervisors were the most often mentioned work resources by employees in the focus groups as well as in previous research [19]. A strong team cohesion, social support, a harmonious and motivating working atmosphere as well as a respectful and appreciative interaction with one another were highlighted in particular. Overall, it became apparent that friendships also developed among work colleagues. Existing studies also highlighted the relevance of appreciation [13, 24] in formal and informal social interaction for employees’ performance and satisfaction [21, 32]. In previous studies social support was able to minimize self-stigmatisation, strengthen employee’s confidence and perceived value [35, 36]. Furthermore, social support to new colleagues had a positive impact on employees’ development, skills and job satisfaction [6, 8, 21, 32]. Just as leisure meetings of employees were evaluated positively in the focus groups, another qualitative study also illustrated how social relationships can be fostered at social events [20]. In general, past research has shown that a supportive work atmosphere may have various positive impacts on social inclusion, sense of belonging and work identity [6, 7, 11, 20, 28, 30]. Likewise, quantitative analysis showed positive impacts of social support on work engagement, productivity, motivation to hold employment, improved skills, self-efficacy and job satisfaction [26, 27, 34, 35], as well as negative associations with self-stigma [35]. Direct supervisor support, e.g. as a mediator in work or team conflicts, was also considered an important resource in the focus groups. The latter were able to build up positive relationships with their employees by means of their appreciative, respectful behaviour and feedback. Our results coincide with previous research as social support from supervisors was stated as a key resource, e.g. in terms of giving feedback [13, 24], promoting acceptance and inclusion, fair leadership [6, 20, 21, 32], establishing a culture of learning from previous mistakes and providing practical assistance [14, 20]. In addition to our results, former studies reported that support from further stakeholders, such as peers, friends, family, mentors or employment specialists, were also recognised as helpful yet less available [6, 13, 22, 24].

Lastly, some aspects of the *work environment* were mentioned as a resource. Thus, appropriate equipment and guidelines as part of occupational health and safety were experienced as beneficial at work. In addition, the provision of snacks in meetings was felt to be appreciative. Previous study results also suggested that materials and equipment enhanced the quality of work life [20].

Overall, our study results on job resources are generally consistent with the current state of research. Therefore, the working conditions for employees with disabilities in social firms are specifically characterised by a high level of social support, flexible work arrangements, structured work activities, individual adjustments of workload as well as the provision of training [19]. In general, the perception and evaluation of certain categories of the GDA criteria [42] is very individual. Thus, aspects of work organisation such as autonomy or flexible working conditions, can be experienced in very different ways depending on employees’ own needs.

### Strengths and limitations

Our study provides several strengths. Given the strong need for research [19], our study provides first empirical findings of employees’ perceived job demands and resources working in social firms in Germany. The qualitative research approach was considered to be the most appropriate for obtaining initial findings in a new field of research [44]. Overall, conducting focus groups in this exploratory study was a suitable method to gain access to this specific target group. This approach allowed for guided group interactions and was particularly appropriate for participants with mental disabilities or hearing impairments. Focus groups were also conducive for enabling internal team exchange on team-related content and work-group representation in exploratory research [45]. Conducting our focus groups, it became apparent that the trust of the employees was particularly gained through the recruitment process. The preceding personal introduction of the researchers and familiarization in the companies therefore may have reduced participation barriers. Likewise, the gatekeeper function of social firms’ supervisors was helpful in increasing participation rate as employees trusted their supervisors. The use of postcards with occupational health and safety related comics as stimulators for each focus group also proved to be conducive for starting conversation with employees. Regarding data analysis, both the analysis and preliminary results were presented and discussed with all authors to strengthen the interpretations and validate the results.

Yet this study has some limitations. Since the qualitative research design aims to gain explorative findings in a new field of research, there is no claim for generalisability of present study results. According to Morgan (1997), the recommended rule of thumb for conducting focus groups is a number of three to five focus groups per research project and a group size of approximately six to ten participants [52], or even three to six participants according to Kitzinger and Barbour [45]. Due to infection prevention reasons during the COVID-19 pandemic, the number of participants was restricted to a maximum of six according to the hygiene coordinator of the research institute. These restrictions were strictly followed by the research team. In order to protect the health and control infection of all participants, a hygienic concept with very high standards and precautions was adopted for the implementation of each focus group. Still, the recommended group size and number of groups was reached in our study. In addition, due to a pandemic-related lockdown in Germany in November and December 2020, two more planned focus groups could not be conducted or rescheduled. The participation of further social firms from other sectors would have been conducive for this study. Moreover, it is not clear whether the restrictions and effects of the COVID-19 pandemic, such as reduced working hours or short-time work [53], had an impact on employees’ experience of job demands and resources. Efforts were made to address these specific strains at the beginning of the focus groups to allow for separate discussion on the general job demands and resources of social firms’ employees beyond the pandemic. Even though the sample was heterogeneous in terms of gender, age, type of disability, and department to uncover different experiences [45], future research should use a broad exploratory approach to focus more on sector-specific differences in terms of job demands and resources.

### Implications for future research

Our study identified that employees primarily experienced job demands regarding work organisation or social relationships. Since previous studies have mainly highlighted the positive influence of social support [8, 11, 13, 20–22, 24, 28, 29], our study results suggest that future research should also investigate in which ways social relationships can be experienced as burdensome and especially what interventions can be implemented in case of interpersonal problems, e.g. communication difficulties. Furthermore, existing research so far only provides evidence that payment and job security have a strong supportive effect for employees [8, 20, 21, 28, 32, 33]. Since our qualitative study showed that the amount of salaries was perceived by some employees as too low for the work performed, future qualitative or quantitative research could conduct international comparisons on perceptions of different forms of remuneration or reward and their influence on health-related outcomes.

With regard to the state of research on job resources in social firms, the focus so far was on work-related outcomes [19]. Future research should therefore investigate qualitatively as well as quantitatively the influence of job resources on health-related outcomes. In this context, future research could also investigate the influence of perceived social support from other stakeholders, e.g. peers, friends, family, mentors or employment specialists, on health-related outcomes. To date, initial evidence on flexible work arrangements has revealed a positive impact on health for employees of social firms [6–8, 13, 14, 20–24, 30–33] as well as on job tenure [24]. Given the results of this study, it is recommended for future research to examine perceptions of working conditions in social firms across various sectors as well as to determine the impact of respective sector-specific working conditions on employees’ health. In this context, further studies on the specific working conditions of social firms are needed in comparison to other companies on the general labour market. Moreover, research should continue on how work tasks can be best adapted to individual competencies, skills and needs of employees with disabilities [8, 14, 31] and how supervisors can be supported in this respect. Another aspect that should be analysed in future research is the assessment of specific challenges and experiences depending on the type of disability.

Since studies on working conditions in social firms have been conducted in a few countries (Canada, Australia, United Kingdom, Italy) so far [19], further studies are needed, e.g. to identify culture-specific and structural differences. With legislative changes of January 1, 2018 in Germany, social firms are required to implement workplace health promotion measures. The present study can serve as a basis for future intervention studies in German social firms for the implementation of health promotion measures. In addition, such intervention studies could, e.g. investigate how effectively employees of social firms can apply coping strategies to deal with certain challenges in their daily work, or assess whether training on team communication and conflict resolution can lead to better collaboration and more positive health outcomes.

### Practical implications

The study results indicate that a variety of different workplace health promotion measures can be implemented in order to maintain and promote employee health at work in social firms. The practical implications for this study are divided into behavioural and structure-related implications [54].

First, practical implications refer to behavioural-related implications addressed to *employees of social firms*. Our findings suggest that employees should be offered training opportunities in which they can strengthen their skills and resources (e.g. own self-care) for dealing with high workloads and time pressure as well as learn communication skills and conflict resolution strategies for healthy collaboration with colleagues at work. For dealing with stressful work tasks or conflict situations, employees could learn mindfulness, the reflection of own needs and expectations as well as coping strategies. In addition, employees should be offered team-building measures to promote healthy cooperation and communication in the team. Awareness of different needs, commonalities and values in the team should be raised.

Second, practical implications refer to structure-related implications concerning *direct supervisors of employees in social firms*. According to current state of research, it is evident that supervisors may influence the health of employees both directly and indirectly, via leadership behaviour, design of working conditions and role model function [55, 56]. Based on our explorative results, it is recommended to offer supervisors further education and training opportunities specific to inclusive workplaces in order to promote appreciative, respectful communication, feedback, and conflict resolution for building trusting, collegial relations with employees. Of particular importance is the promotion of healthy team cooperation and communication by the supervisor. It is recommended to offer social support for conflicts among colleagues or problems at work as well as to introduce regular team meetings and to establish a harmonious work atmosphere including a culture of accepting failures. In addition, supervisors are able to influence the working conditions of employees in a health promoting way. In social firms, supervisors should pay particular attention to ensure that working conditions (in terms of work content, work organisation, work environment) are adapted to individual needs and are not experienced as burdensome by employees. Among other things, attention should be paid here to flexible work planning, organisation and break scheduling, a balance of (un)desired tasks, task variety, job training and a moderate workload.

Third, *management of social firms* should be addressed in terms of structure-related implications in order to reduce stressful working conditions at the workplace. Our findings suggest social firms to generally strive for a long-term personnel planning with low staff turnover or staff shortage, for a fair remuneration system and actively work on a value-oriented and open corporate culture in order to strengthen respect, acceptance and tolerance among staff. Furthermore, it is recommended to create structures and opportunities for staff participation, e.g. in decision-making processes, as well as to provide training and development, promotion and career trajectories for employees and supervisors. These measures may improve the inclusion of people with disabilities in the general labour market and provide support for everyday challenges in the workplace. Lastly, with regard to the working environment, it should be ensured that employees are not exposed to stressful environmental conditions, such as high levels of noise, and that a comprehensive occupational health and safety approach is followed.

Table 4 displays practical implications for employees of social firms based on present study results.

**Table 4:** Practical implications for employees, supervisors and management of social firms

Behavioural-related implications	Structural-related implications
*Employees:* • Training to strengthen own resources in self-care (mindfulness, self-reflection, coping strategies) • Training for communication and conflict resolution • Team-building measures to promote healthy cooperation (raising awareness of different needs, commonalities and values)	*Supervisors:* • Building trusting, collegial relationships with employees • Training for appreciative, respectful communication, feedback and conflict resolution • Mediation / social support for conflicts among colleagues or problems at work • Fostering collegial teamwork (regular team meetings, structured work processes, harmonious work atmosphere) • Flexible adaptation of working conditions to the individual needs of employees (concerning work content, work organisation, work environment)
	*Management:* • Long-term personnel planning (low staff turnover, staff shortage) • Fair remuneration system • Value-oriented corporate culture, open culture of discussion and feedback • Opportunities for staff participation • Provide support opportunities for employees and supervisors (training and development, promotion and career trajectories) • Follow a comprehensive occupational health and safety approach

## Conclusions

Our study was the first to assess working conditions of employees with disabilities in German social firms according to the JD-R model [38]. By using a qualitative research approach, we identified and categorized job demands and resources based on the criteria of the Joint German Occupational Safety and Health Strategy (GDA) work content, work organisation, social relations and work environment [42]. The focus group results indicated that job demands were experienced particularly in terms of work organisation, whereas social relationships to colleagues and supervisors were experienced as a central resource. This study provides a basis for further qualitative and quantitative studies that examine, among other things, sector- and task-specific working conditions in social firms. Based on our findings regarding working conditions in social firms, practical implications are presented. The present study can serve as a basis for future intervention studies in German social firms for the development, implementation and evaluation of workplace health promotion measures.

## Supplementary Information

**Additional file 1.** COREQ-Checklist. This document contains a checklist of items that were used for reporting the study design and results.

## Data Availability

The datasets analysed during the current study are not publicly available due to German national data protection regulations but are available from the corresponding author on reasonable request.
